# On the use of abiotic sialic acids to attenuate cell inflammation

**DOI:** 10.1038/s41598-018-35477-2

**Published:** 2018-11-23

**Authors:** Zhongwei Xue, Hu Zhao, Rui Zhu, Congcong Chen, Hongzhi Cao, Jiahuai Han, Shoufa Han

**Affiliations:** 1 Department of Chemical Biology, College of Chemistry and Chemical Engineering, State Key Laboratory for Physical Chemistry of Solid Surfaces, the Key Laboratory for Chemical Biology of Fujian Province, The MOE Key Laboratory of Spectrochemical Analysis & Instrumentation, and Innovation Center for Cell Biology, Xiamen University, Xiamen, 361005 China; 2 State key Laboratory of Cellular Stress Biology, Innovation Center for Cell Biology, School of Life Sciences, Xiamen University, Xiamen, 361005 China; 3 National Glycoengineering research center, Shandong University, Jinan, 250012 China

## Abstract

Sialic acid (Sia) residues on cell surface are critical for myriad cellular events such as immunity and inflammation. We herein reported the use of abiotic Sia to raise the thresholds of inflammatory cell responses. Identified from a panel of structurally diversified Sia analogs via a cell inflammation assay, Sia-2, with *N*-butyryl moiety at C-5, markedly lowered LPS-stimulated NF-κB activity in macrophages. Further analysis shows that Sia-2 attenuates phosphorylation of IκB and Erk1/2/p38/JNK, critical for NF-κB signaling and MAPK signaling, and lowers gene transcription of proinflammatory interleukin-6. These results support the use of abiotic Sia as promising agents to modulate cell surface Sia-pertinent cell signaling.

## Introduction

Inflammation triggered by harmful stimuli leads to activation of cell surface receptors such as Toll-like receptors (TLRs). Activation of TLR4 by lipopolysaccharide (LPS) gives rise to successive downstream signaling events in cells and eventually production of proinflammatory cytokines^1,2^. As excess cytokines cause endotoxic shock and death, approaches attenuating cellular inflammation are of therapeutic potentials.

Sialic acids (Sia) is a family of natural derivatives of N-acetyl-neuraminic acid (NeuAc)^3,4^, and is the common terminal residues of cell surface glycans. Cell surface Sia underlies diverse biological events ranging from cell adhesion, immunity, to inflammation^5,6^. Positioned at the outermost glycocalyx, Sia residue is prone to bind receptors on the same cell (“in cis”) or on the apposing cells (“in trans”)^7–9^, and also prone to desialylation^10–13^. The Sia dynamics are critical for the cognate biological functions. For instance, cell inflammatory responses were increased upon removal of Sia from cell surface ligands or receptors^14–17^. Particularly desialylation of TLR4 ligands was required for TLR4 signaling^18^ and LPS induced cytokine production in dendritic cells^19^. These reports clearly show Sia functions as a negative regulator of cell inflammation and particularly LPS/TLR4 signaling events.

Sia derivatives have been metabolically incorporated into cell surface glycoconjugates with supplemented *N*-acyl mannosamines^20–24^ or synthetic abiotic Sia^25–27^. Given the anti-inflammatory roles of Sia^14–19^, we set to explore the use of structurally modified Sia to raise the thresholds of cellular inflammation. Herein, a panel of structurally diversified Sia derivatives were synthesized and screened by effects on LPS/TLR4 triggered nuclear factor-κB (NF-κB) signaling, which has been linked to diverse pathological conditions such as cancer and inflammation^28–30^. Sia-2 was identified to effectively inhibit NF-κB signaling and MAPK signaling and gene transcription of proinflammatory IL-6 in LPS-stimulated macrophages.

## Results and Discussion

**Figure 1:**
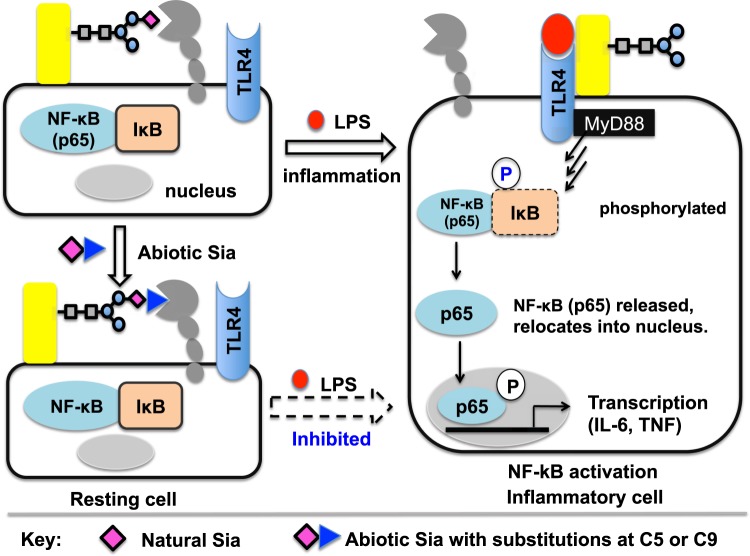
Schematic for altering NF-κB signaling with abiotic Sia. NF-κB signaling features p65/IkB phosphorylation and gene transcription of proinflammatory cytokines. Anti-inflammatory abiotic Sia, proposed via altered cis binding of TLR-4 ligand, lowers phosphorylation of p65/IkB and transcription of IL-6 gene in NF-κB signaling.

Given desialylation enhanced inflammation^14–17^ and the vital roles of Sia *in cis* for immune cell activity^31,32^, we envisioned that abiotic Sia with appropriate chemical groups might alter cell inflammation responses, possibly by altered affinity on *cis* binding (Fig. 1). Rational design of Sia analogs with desired properties is limited by the complex and multiple Sia interactions on cell surface. Inspired by the use of glycan microarray to identify high affinity ligands of sialic acid-binding immunoglobulin-like lectins (Siglecs) from substituted sialosides^33,34^, we implemented an *in vitro* cell reporter assay to screen abiotic Sia capable of lowering cell inflammation.

### An ***in vitro*** cell based assay to monitor NF-κB signaling

**Figure 2:**
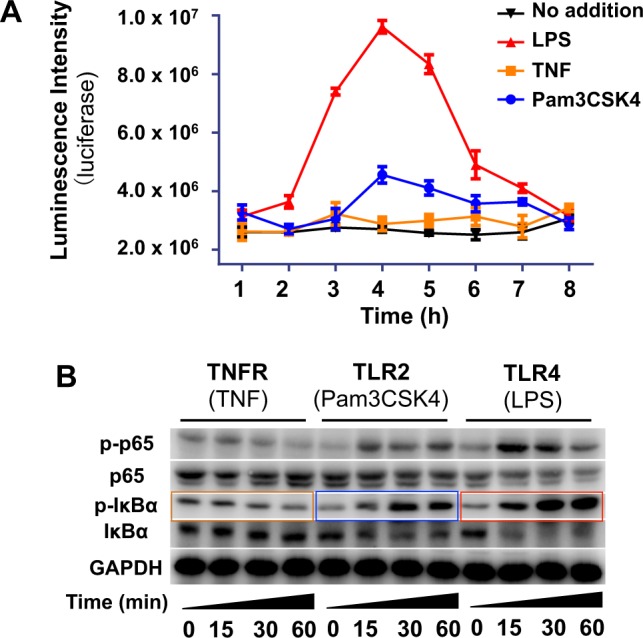
LPS mediated NF-κB signaling in NF-κB/Luc^+^ Raw 264.7 cells. NF-κB/Luc^+^ cells were treated with LPS, TNF or Pam3CSK4 and then lysed. Cell lysate was measured for luciferase activity using a Luciferin-ATP bioluminescence protocol (**A**) or analyzed by Western blotting to probe the levels of phosphorylated p65 (p-p65) and p-IκBα (**B**). Error bars stands for stand error of mean on results from 5 samples.

NF-κB is sequestered in the cytosol by inhibitory IκB proteins in resting cells, while phosphorylation of IκB in inflamed cells leads to relocation of NF-κB from cytosol into cell nucleus, and induces transcription of proinflammatory genes (Fig. 1)^35^. To assay LPS/TLR4-mediated inflammation, Raw 264.7 cells were transfected to introduce firefly luciferase gene transcriptionally controlled by NF-κB (NF-κB/Luc^+^), allowing NF-κB signaling monitored by luciferase activity. Apart from TLR4, NF-κB can also be activated by several cell surface receptors such as TLR2 and the tumor necrosis factor (TNFR)^35^. As such NF-κB/Luc^+^ cells were stimulated with LPS, Pam3CSK4 (angonist of TLR2) or Tumor Necrosis Factor-α (TNF) specific for TNFR, respectively, and then assayed for the levels of NF-κB signaling. NF-κB/Luc^+^ cells exhibited markedly and transiently enhanced luciferase activity peaked at 4 h post LPS stimulation whereas low to moderate luciferase activity was induced by TNF or Pam3CSK4 (Fig. 2A). Consistently, Western blot analysis revealed higher levels of phosphorylated IκB-α (p-IκBα) and p-p65 induced by LPS over TNFR or TLR2 (Fig. 2B). These data validate the use of NF-κB/Luc^+^ cells to monitor LPS/TLR4/NF-κB signaling by NF-κB driven transcription of luciferase and to screen anti-inflammatory abiotic Sia.

### Abiotic Sia screening by altered NF-κB signaling

**Figure 3:**
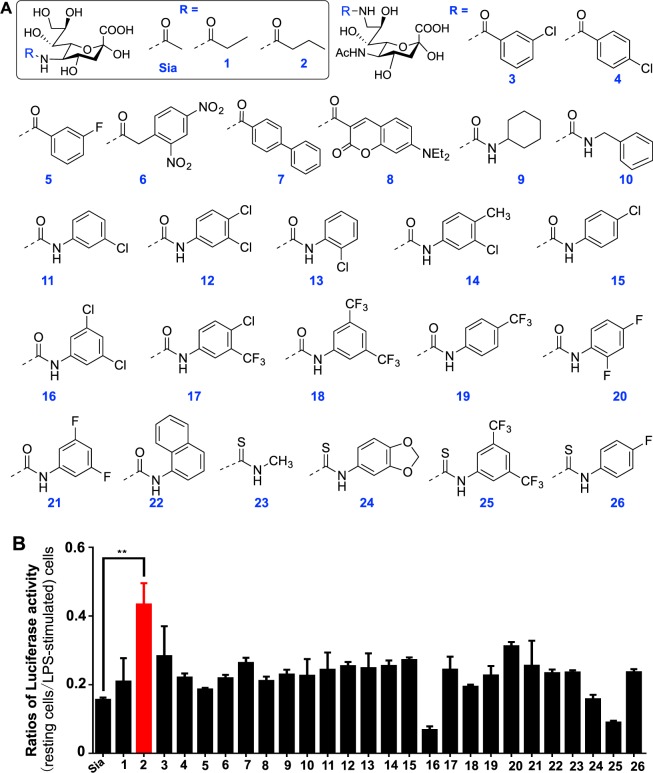
Effects of abiotic Sia on NF-κB signaling activity. (**A**) Chemical structures of the abiotic Sia tested. Sia analogs (Sia-1/Sia2) carry substitutions at C-5 and are shown in the rectangle, while the rest analogs (Sia3-Sia-26) bear substitutions at C-9. (**B**) Differential effects of abiotic Sia on NF-κB activity. NF-κB/Luc^+^ cells were incubated with individual abiotic Sia (0.5 mM) for 24 h, and then stimulated with or without LPS. The cells were lysed and the lysate were measured for luciferase activity. The ratio of luciferase in LPS^-^ cells (restive cells) over LPS^+^ cells (inflamed) were recorded. Error bars stands for stand error of mean on results from 5 samples.

To discern abiotic Sia on cell inflammation, NF-κB/Luc^+^ cells were cultured with a panel of structurally diversified Sia (Fig. 3A), stimulated with LPS, and then measured for the corresponding luciferase activity. Cells treated natural Sia were used as the control. The ratios of luciferase activity in LPS-free cells (restive cells) over LPS-stimulated cells (inflamed) were used to indicate the efficacy of abiotic Sia on cell inflammation. Sia-2, with *N*-butyryl group at C5, was identified to attenuate LPS-stimulated NF-κB activity in cells (Fig. 3B). As high ratios indicate lowered NF-κB signaling in LPS^+^ cells, these results suggest anti-inflammatory effects of Sia-2. In contrast, treatment with Sia-16 and Sia-25, sharing a 3, 5-disubstituted phenyl moiety at C-9, gave rise to much lower ratios of luciferase activity in resting cells over LPS-stimulated cells (Fig. 3B), showing that these analogs potentiated NF-κB activation in LPS^+^ cells. In addition, no detrimental effects of Sia-2 were observed on cell viability and proliferation, which is beneficial for therapeutic application. These results validate the feasibility to alter cell inflammation with structurally modified Sia.

### Molecular mechanism of inhibiting NF-κB and MAPK signaling by Sia-2

**Figure 4:**
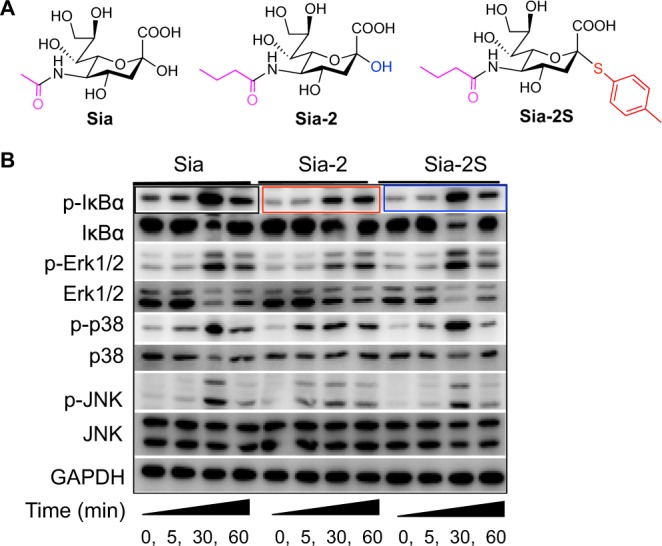
Inhibitory effects of Sia-2 on phosphorylation of key components of inflammation-relevant cell signaling pathways. Raw 264.7 cells were incubated with Sia-2, Sia-2S, or Sia (0.5 mM), stimulated with LPS for varied periods of time (0, 5, 30, 60 min) and then lysed. The levels of phosphorylated proteins in cell lysate were examined by Western blotting as a function of LPS-stimulation time.

We proceeded to assess the structural factors of Sia-2 critical for the observed anti-inflammatory effects. We synthesized thioglycoside of Sia-2 (Sia-2S), which differs from Sia-2 in the lack of C-2 hydroxyl group (Fig. 4A). Raw 264.7 cells were cultivated with Sia, Sia-2 or Sia-2S, respectively, and then examined for phosphorylation of key protein components of LPS-TLR4/NF-κB signaling. Western Blotting analysis shows that the levels of p-IκBα (phosphorylated IκBα), dramatically induced at 30 min and substantially decayed by 60 min post LPS-stimulation in Sia^+^ cells (Fig. 4B) in Sia-treated cells, are markedly lowered in Sia-2^+^ cells at 0–30 min and remained largely unchanged up to 60 min after LPS-stimulation. Given the critical of p-IκBα for NF-κB signaling activity, the attenuated phosphorylation of IκBα by Sia-2 is in line with the inhibitory effect of Sia-2 on NF-κB signaling observed in NF-κB/Luc^+^ cells (Fig. 3B). In contrast with the attenuated p-IκBα in Sia-2^+^ cells, the level of p-IκBα in Sia-2S^+^ cells resembles that in Sia^+^ cells, showing the essential roles of C-2 hydroxyl group and N-butyryl moiety of Sia-2 for inhibiting cell inflammation. C-2 hydroxyl group is essential for enzymatic activation of Sia into cytidine-5′-monophosphate-Sia (CMP-Sia) in the presence of cytidine triphosphate for cell sialylation^3^. Sia-2S with C-2 hydroxyl group masked is unable to be incorporated into cell surface. The differential effects of Sia-2 and Sia-2S on NF-κB signaling strongly support that Sia-2 is metabolically installed on cell surface in the observed dampened LPS/NF-κB signaling as outlined in Fig. 1.

Mitogen-activated protein kinases (MAPK) are critical for cell proliferation^36–39^, and are activatable to TLR4^1,2^. In addition, desialylation activate ERK1/2 kinases and enhance cytokine production in monocytes^40^, suggesting the involvement of cell surface sialoglycoconjugates in MAPK signaling. We thus evaluated the effects of Sia-2 on MAPK signaling pathways comprising the extracellular signal-regulated kinase (ERK) family, p38 kinase family, and c-Jun N-terminal kinase family (JNK). p-Erk1/2, p-p38, and p-JNK is obviously lowered in LPS^+^ cells treated with Sia-2 over Sia-2S or Sia (Fig. 4B), strongly indicating that Sia-2 could affects distinct cell signaling pathways that necessitate cell surface Sia for signal transduction from cell surface to cell interior.

### Effects of Sia-2 on IL-6 transcription and translation

**Figure 5:**
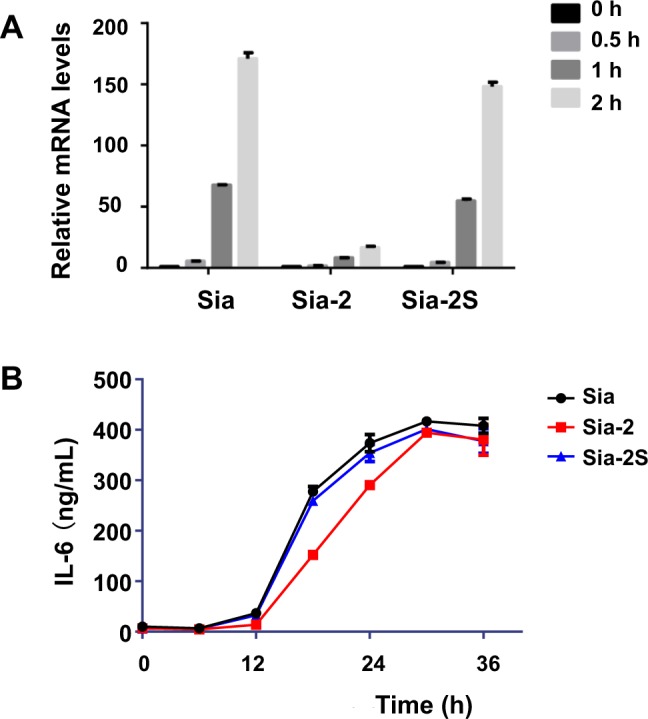
Differential effects of Sia-2 and Sia-2S on gene transcription and translation of IL-6. (**A**) Raw 264.7 cells were incubated with Sia-2, Sia-2S, or Sia (0.5 mM), stimulated with LPS for varied periods of time (0, 0.5, 1, 2 h), and then quantitated for IL-6 mRNA levels by qRT-PCR, which were normalized to m-GAPDH. (**B**) Raw 264.7 cells cultivated with Sia-2, Sia-2S, or Sia were respectively stimulated with LPS. The cells were maintained in fresh medium for varied periods of time (0–36 h). The levels of IL-6 excreted into extracellular medium were quantitated by ELISA assay at indicated time points.

With the defined inhibition on phosphorylation of key proteins in cell inflammation signaling, we continued to determine the influence of Sia-2 on down-stream gene transcription of proinflammatory cytokines. Quantitative real time polymerase chain reaction (qPCR) analysis showed that the levels of IL-6 mRNA rise dramatically at 1–2 h post LPS-stimulation in control cells (Fig. 5A), which is consistent with NF-κB activation triggered gene transcription^29^. In sharp contrast, Sia-2 treatment leads to 10-fold decrease in the levels of IL-6 mRNA in LPS^+^ cells whereas Sia-2S exhibited no obvious effects on IL-6 mRNA transcription, demonstrating the inhibition of gene transcription in LPS/NF-κB signaling by Sia-2. Next, we quantitated IL-6 excreted into extra-cellular medium from inflamed cells as a consequence of sugar-inhibited gene transcription. We observe higher levels of IL-6 from Sia- or Sia-2S-treated cells than Sia-2^+^ cells at 30–36 h post LPS-stimulation (Fig. 5B), showing that Sia-2 could lower IL-6 translation relative to Sia and Sia-2S.

Diet-derived nonhuman N-glycolylneuraminic acid (NeuGc) as been confirmed to be incorporated into tumors and contributes to inflammatory responses^41,42^. In addition, Sia with varied substitutions have been metabolically introduced into cell surface glycans for cancer immunotherapy^26,43–46^. To the best of our knowledge, abiotic Sia with inhibitory effects on cell inflammation have been unreported. Herein, we presented several lines of findings that abiotic Sia-2 dampens inflammatory NF-κB activity, downstream gene transcription and translation of proinflammatory IL-6. These results support the use of abiotic Sia as novel functional probes to modulate cell inflammation.

## Conclusions

Cell surface-exposed Sia residues participate in a plethora of cellular events whereby desialylatioan and cis-binding reciprocally modulate upstream cell inflammatory responses. Targeting aberrant NF-κB activation in autoimmunity and tumorigenesis, we herein report the identification of anti-inflammatory Sia-2 from a panel of structurally diversified abiotic Sia analogs. Sia-2 attenuates LPS/NF-κB signaling by defined effects on multiple signaling stages including IκBα phosphorylation, gene transcription and protein translation. These finding show that abiotic Sia with appropriate substitutions offers a new route to modulate cellular inflammation and possibly broad cell signaling events pertinent to Sia residues on cell surface.

## Experimental Procedure

### Materials and method

**Figure 6:**
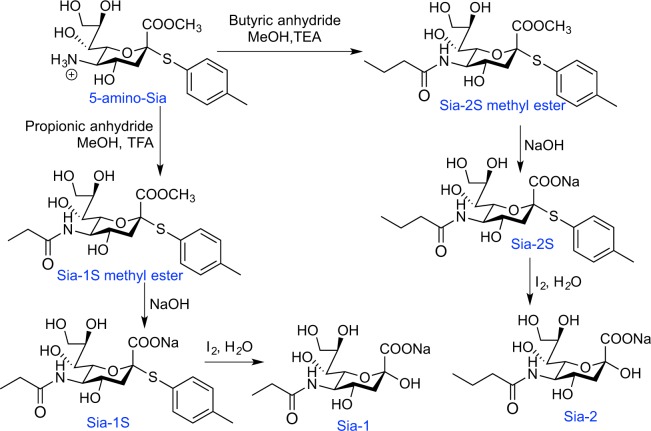
Synthetic route of Sia-1 and Sia-2.

**Figure 7:**
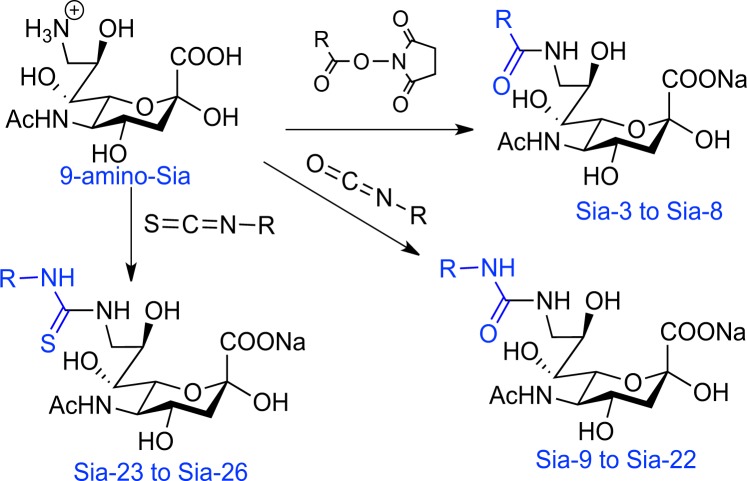
Synthetic routes of C-9 N-acylated abiotic Sia.

Lipopolysaccharide (LPS) was purchased from Sigma. Antibodies were purchased from cell Signaling Technology. Column chromatography was performed on silica gel (100–200 mesh). NMR spectra were recorded on a Bruker instrument using tetramethyl silane as the internal reference. Mass analysis was performed in Bruker En Apex ultra 7.0 T FT-MS. Reported procedures were employed to synthesize 5-amino-Sia-S, (Fig. 6)^47^, 9-amino-9-deoxy Sia (9-amino-Sia, Fig. 7)^25^ and Sia-7^48^. All other chemicals were used as received from Alfa Aesar.

The following antibodies (for p-P65: phospho-NF-κB p65 (Ser536) (93H1) Rabbit mAb #3033, for P65: NF-κB p65 (D14E12) XP® Rabbit mAb #8242; for p-IκBα: Phospho-IκBα (Ser32/36) (5A5) Mouse mAb #9246; for IκBα: IκBα Antibody #9242) were purchased from Cell Signaling Technology. GAPDH antibody was purchased from Proteintech. HRP-conjugated secondary antivody for p-P65/P65 (Goat anti-Rabbit IgG (H + L)), and for p-IκBα/IκBα and GAPDH (Goat anti-Mouse IgG sencondary antibodies (H + L)) were obtained from ThermoFisher.

293 T and Raw 264.7 cells were obtained from American Type Culture Collection (ATCC). Cells were maintained in Dulbecco’s modified Eagle’s medium (DMEM), supplemented with 10% fetal bovine serum, 2 mM L-glutamine, 100 IU penicillin, and 100 mg/mL streptomycin at 37 °C in a humidified incubator containing 5% CO_2_. Flow cytometry analysis was performed on BD Fortessa, the fluorescence emission intensity of F-SNA was recorded by FITC filter (500–560 nm) using excitation wavelength of 488 nm. 10000 cells were gated under identical conditions, analyzed and the data were processed by GraphPad Prism5. Confocal fluorescence microscopic imaging was performed on Zeiss LSM 780 using the following filters: λex = 488 nm and λem = 499–553 nm for F-SNA. The fluorescence of F-SNA in cells was shown in green. Fluorescense images were merged using Photoshop CS6.

Construction of NF-κB/Luc^+^ Raw 264.7 responsive to LPS: The NF-κB luciferase reporter gene was cloned into BamHI and XhoI sites of the lentiviral vector pBOB using the Exo III-assisted ligase-free cloning method as described^49^. All plasmids were verified by DNA sequencing. For lentivirus production, HEK293T cells were transfected by the calcium phosphate precipitation method. The virus-containing medium was harvested 36–48 h later and was added to Raw 264.7 cells. Then we selected the high-response single clone (NF-κB/Luc^+^ Raw 264.7) for experiments. NF-κB/Luc^+^ Raw 264.7 cells were treated with LPS (100 ng/mL), TNF (100 ng/mL) or Pam3CSK4 (200 ng/mL) for different times (1, 2, 3, 4, 5, 6, 7, and 8 h)”. The cells were homogenized and the lysate was measured for detection of luciferase activity using ONE-Glo™ EX Luciferase Assay System (Promega) according to manufacturer’s recommendations.

Screening of anti-inflammatory abiotic Sia by NF-κB/Luc+ Raw 264.7: NF-κB/Luc+ Raw 264.7 cells were cultured in DMEM contained different sialic acid derivatives (0.5 mM) for 24 h and then stimulated with 100 ng/mL LPS for 4 h. Cells were measured for luciferase activity using ONE-Glo™ EX Luciferase Assay System (Promega) according to manufacturer’s recommendations.

Western blot analysis: Raw 264.7 cells were first cultured for 24 h in DMEM spiked with Sia (0.5 mM), Sia-2 (0.5 mM) or Sia-2S (0.5 mM) and stimulated with LPS (100 ng/mL) for different times (0, 5, 30, and 60 min). The cell samples were respectively added to 1.2 x SDS-PAGE loading buffer, resolved on 10% SDS-PAGE gels, transferred to nitrocellulose, and blocked with 5% bovine serum albumin in PBST (Dulbecco’s Phosphate Buffered Saline with 0.05% Tween-20) for 1 h at room temperature. The blocked membrane was incubated with specific first antibodies in blocking buffer overnight at 4  C and washed with PBST (3 × 10 min per wash). Followed by HRP-conjugated second antibodies in blocking buffer for 1 h at room temperature. Then the membrane was washed with PBST (3 × 10 min per wash), and developed using Immobilion Western Chemilum HRP Substrate (Merck).

Detection of mRNA IL6 was performed following reported procedures^50,51^: Raw 264.7 cells were first cultured for 24 h in DMEM spiked with Sia (0.5 mM), Sia-2 (0.5 mM) or Sia-2S (0.5 mM) and stimulated with LPS (100 ng/mL) for different times (0, 0.5, 1, and 2 h). Total RNA of stimulated cells was obtained from stimulated Raw 264.7 cells by RNA-iso reagent (TakaRa). Purified RNA was treated with RNAse-free DNAse (Thermo Fisher Scientific) for 2 hours at 37 °C. The DNAse was then inactivated by the addition of 2.5 mM EDTA and incubation of the samples at 60 °C for 10 minutes. Total RNA (2 mg) was reverse-transcribed to cDNA using Random Hexamer Primers (Thermo Fisher Scientific) and M-MLV reverse transcription (BGI, Shenzhen, China). The levels of IL-6 and beta-actin were determined by SYBR-Green I real time quantitative PCR in a CFX96 real-time RT-PCR detection system (Bio-Rad). PCR amplification was carried out over 40 cycles using the following conditions: denaturation at 95 °C for 20 seconds, annealing at 58 °C for 20 seconds, and elongation at 72 °C for 20 seconds. Primers of mouse GAPDH are 5′-TGTGTCCGTCGTGGATCTG-3′ and 5′-CCTGCTTCACCACCTTCTTGA-3′. Primers of mouse IL-6 are 5′-TCCATCCAGTTGCCTTCTTG-3′ and 5′-GGTCTGTTGGGAGTGGTATC -3′.

ELISA analysis: Raw 264.7 macrophages were cultured in 24-well plate with or without different sialic acid derivatives (0.5 mM) for 24 h, and then incubated with 100 ng/mL of LPS for 0, 6, 12, 18, 24, 30, and 36 h. IL-6 in the culture medium were determined by ELISA kit (eBioscience™ Mouse IL-6 ELISA Ready-SET-Go!™ Kit) according to the manufacturer’s recommendations. IL-6 was measured in triplicate, and the ELISA plates were read using a microplate reader (Molecular Devices Spectra MAX).

Cell proliferation and cytotoxicity assay: The cytotoxicity of different sialic acid derivatives was evaluated on Raw 264.7 cells. The cells were cultured with medium containing each of the Sia derivatives (0.5 mM) for 0, 12, and 24 h. The cell number and cell viability were determined using the CellTiter 96® AQueous One Solution Cell Proliferation Assay (Promega) according to the manufacturer’s recommendations.

### Synthesis of C5 N-acylated Sia

To the solution of 5-amino-9-deoxy Sia (4 g) in methanol (50 mL) on ice was added TEA (10 mL) followed by stepwise addition of propionic anhydride (5 mL) or butyric anhydride (5 mL). The solution was stirred at room temperature for 20 min, and concentrated. The residues were purified by column chromatography using EtOAc-EtOAc/MeoH (5:1) as the eluent to give Sia-1S methyl ester (4.1 g, 90%) or Sia-2S methyl ester (4.3 g, 91%) as off-white solid **(**Fig. 5**)**.

Sia-1S methyl ester or Sia-2S methyl ester (2 g) were respectively dissolved in methanol (20 mL). To the above solutions were respectively added aqueous sodium hydroxide (3 M, 7 mL) and stirred at room temperature for 1 hour. The solutions were adjusted to pH 6 with addition of hydrochloric acid solution (1 M), and then concentrated. The residue was purified by column chromatography using DCM/CH_3_OH/AcOH: 50/5/1 as the eluent to give Sia-1S (1.73 g, 89%) or Sia-2S (1.69 g, 87%) as off-white solid. Sia-1S or Sia-2S (1 g) was respectively dissolved in water (30 mL). To the above solutions was respectively added iodine (1.1 eqv.). The reaction was stirred for 12 h at which time TLC indicated the reaction was complete. The reaction was extracted with CH_2_Cl_2_ (8 × 20 mL) and the aqueous layer was concentrated to give Sia-1 (0.7 g, 92%) or Sia-2 (0.69 g, 91%) as the desired products.

### Synthesis of C9-N-substituted Sia

To the solution of 9-amino-9-deoxy Sia (1 g) in dixoane/water (30 mL, 2:1) was respectively added the corresponding isocyanate, cyanate or acyl NHS ester (2 equiv.). The solution was adjusted to pH 9 with addition of saturated sodium carbonate and then stirred at room temperature for 12 h. The solvent was removed under reduced pressure and the residue was purified by silica gel chromatography and C-18 reverse phase chromatography to give the C-9 substituted Sia in 30–70% yields **(**Fig. 7**)**.

### Sia-1S methyl ester

^1^H NMR (500 MHz, CD_3_OD) δ 7.46 (d, *J* = 7.9 Hz, 2 H), 7.22 (d, *J* = 7.8 Hz, 2 H), 3.86–3.78 (m, 3 H), 3.68 (s, 3 H), 3.67–3.58 (m, 2 H), 3.48 (d, *J* = 8.8, 1.9 Hz, 1 H), 3.39 (d, *J* = 10.4, 1.8 Hz, 1 H), 2.88 (dd, *J* = 12.9, 4.7 Hz, 1 H), 2.39 (s, 3 H), 2.29 (q, 2 H), 1.88 (dd, *J* = 12.9, 11.4 Hz, 1 H), 1.16 (t, *J* = 7.6 Hz, 3 H). ^13^C NMR (126 MHz, CD_3_OD) δ 177.59, 169.74, 140.38, 136.42, 129.26, 125.27, 86.56, 76.06, 71.56, 68.75, 67.55, 63.07, 52.08, 51.92, 40.39, 28.73, 19.93, 8.86. MS (C_20_H_29_NO_8_S): calculated (MS + Na): 446.15, found: 445.98.

### Sia-1S

^1^H NMR (500 MHz, Methanol-*d*_4_) δ 7.52 (d, *J* = 8.0 Hz, 2 H), 7.12 (d, *J* = 7.8 Hz, 2 H), 3.92–3.85 (m, 1 H), 3.82 (dd, *J* = 11.4, 2.7 Hz, 1 H), 3.75 (dt, *J* = 11.1, 9.8, 4.8 Hz, 1 H), 3.68–3.60 (m, 2 H), 3.53–3.43 (m, 2 H), 2.90 (dd, *J* = 12.4, 4.9 Hz, 1 H), 2.33 (s, 3 H), 2.26 (q, *J* = 7.6 Hz, 2 H), 1.65 (d, *J* = 12.4, 11.1 Hz, 1 H), 1.14 (t, *J* = 7.6 Hz, 3 H). ^13^C NMR (126 MHz, CD_3_OD) δ 177.94, 173.08, 138.57, 135.98, 128.71, 127.70, 88.76, 75.29, 71.68, 69.04, 68.22, 63.11, 52.50, 41.27, 28.65, 19.90, 8.98. MS (C_19_H_27_NO_8_S): calculated (MS + Na): 452.13, found: 451.97.

### Sia-1

^1^H NMR (500 MHz, D_2_O) δ 4.10–3.99 (m, 2 H), 3.93 (t, *J* = 10.2 Hz, 1 H), 3.85 (d, *J* = 11.8, 2.7 Hz, 1 H), 3.78 (dd, *J* = 9.0, 6.4, 2.6 Hz, 1 H), 3.67–3.59 (m, 1 H), 3.52 (d, *J* = 9.0 Hz, 1 H), 2.33 (q, 2 H), 2.24 (dd, *J* = 13.0, 4.9 Hz, 1 H), 1.85 (t, *J* = 12.3 Hz, 1 H), 1.15 (t, *J* = 7.7 Hz, 3 H). ^13^C NMR (126 MHz, D_2_O) δ 178.77, 176.72, 96.42, 70.36, 70.25, 68.53, 67.12, 63.24, 52.12, 39.44, 29.34, 9.60. HRMS (C_12_H_21_NO_9_): calculated (MS-H): 322.11435, found: 322.11415.

### Sia-2S methyl ester

^1^H NMR (500 MHz, CD_3_OD) δ 7.46 (d, *J* = 7.9 Hz, 2 H), 7.22 (d, *J* = 7.8 Hz, 2 H), 3.88–3.78 (m, 3 H), 3.68 (s, 3 H), 3.67–3.59 (m, 2 H), 3.49 (dd, *J* = 8.7, 1.8 Hz, 1 H), 3.38 (dd, *J* = 10.5, 1.8 Hz, 1 H), 2.88 (dd, *J* = 12.9, 4.8 Hz, 1 H), 2.39 (s, 3 H), 2.25 (td, *J* = 7.3, 3.2 Hz, 2 H), 1.88 (dd, *J* = 12.9, 11.4 Hz, 1 H), 1.67 (h, *J* = 7.4 Hz, 2 H), 0.98 (t, *J* = 7.4 Hz, 3 H). ^13^C NMR (126 MHz, CD_3_OD) δ 178.15, 171.13, 141.77, 137.81, 130.65, 126.66, 87.95, 77.48, 72.96, 70.21, 68.92, 64.50, 53.49, 53.32, 41.83, 38.97, 21.33, 20.23, 14.03. MS (C_21_H_31_NO_8_S): calculated (MS + Na): 480.17, found: 479.88.

### Sia-2S

^1^H NMR (500 MHz, CD_3_OD) δ 7.51 (d, *J* = 7.8 Hz, 2 H), 7.14 (d, *J* = 7.8 Hz, 2 H), 3.86 (ddd, *J* = 8.7, 5.5, 2.5 Hz, 1 H), 3.82 (dd, *J* = 11.4, 2.6 Hz, 1 H), 3.75 (td, *J* = 10.3, 4.6 Hz, 1 H), 3.71–3.64 (m, 1 H), 3.61 (dd, *J* = 11.4, 5.6 Hz, 1 H), 3.47 (ddd, *J* = 9.6, 6.9, 1.9 Hz, 2 H), 2.90 (dd, *J* = 12.6, 4.7 Hz, 1 H), 2.34 (s, 3 H), 2.24 (td, *J* = 7.3, 2.2 Hz, 2 H), 1.75–1.68 (m, 1 H), 1.65 (p, *J* = 7.4 Hz, 2 H), 0.96 (t, *J* = 7.4 Hz, 3 H). ^13^C NMR (126 MHz, CD_3_OD) δ 177.08, 139.11, 136.08, 128.89, 126.99, 75.51, 71.73, 69.03, 67.97, 63.06, 52.44, 41.02, 37.51, 19.95, 18.91, 12.67. MS (C_20_H_29_NO_8_S): calculated (MS + Na): 466.15, found: 465.93.

### Sia-2

^1^H NMR (500 MHz, D_2_O) δ 4.17–4.03 (m, 2 H), 4.01–3.92 (m, 1 H), 3.89–3.84 (m, 1 H), 3.79 (ddd, *J* = 9.2, 6.3, 2.7 Hz, 1 H), 3.68–3.54 (m, 2 H), 2.37–2.24 (m, 3 H), 1.90 (q, *J* = 12.3, 10.9 Hz, 1 H), 1.65 (h, *J* = 7.7 Hz, 2 H), 0.95 (t, *J* = 7.3 Hz, 3 H). ^13^C NMR (126 MHz, D_2_O) δ 178.53, 174.66, 96.20, 71.03, 70.87, 68.95, 67.26, 63.75, 52.58, 39.69, 38.53, 19.63, 13.48. HRMS (C_13_H_23_NO_9_): calculated (MS-H): 336.13000, found:336.12973.

### Sia-3

^1^H NMR (500 MHz, D_2_O) δ 7.72 (s, 1 H), 7.62 (d, *J* = 8.5 Hz, 1 H), 7.57 (d, *J* = 7.9 Hz, 1 H), 7.45 (t, *J* = 7.8 Hz, 1 H), 4.03 (d, *J* = 10.5 Hz, 2 H), 3.92 (m, *J* = 18.3, 8.3 Hz, 2 H), 3.75 (d, *J* = 12.1 Hz, 1 H), 3.49 (m, *J* = 11.6, 7.6 Hz, 2 H), 2.23 (dd, *J* = 12.9, 4.6 Hz, 1 H), 2.03–2.00 (s, 3 H), 1.85 (t, *J* = 12.2 Hz, 1 H). ^13^C NMR (214 MHz, CD_3_OD) δ 174.46, 169.24, 137.69, 135.57, 133.32, 132.51, 131.18, 130.99, 130.48, 128.93, 128.55, 126.72, 71.94, 71.82, 70.73, 68.58, 54.11, 45.10, 41.62, 30.73, 22.80. HRMS (C_18_H_23_ClN_2_O_9_): calculated (MS-H): 445.10138, found: 445.10144.

### Sia-4

^1^H NMR (500 MHz, D_2_O) δ 7.73 (d, *J* = 8.4 Hz, 2 H), 7.53 (d, *J* = 8.4 Hz, 2 H), 4.03 (d, *J* = 16.9, 7.5 Hz, 2 H), 3.96–3.88 (m, 2 H), 3.76 (d, *J* = 14.1, 2.8 Hz, 1 H), 3.55–3.47 (m, 2 H), 2.22 (dd, *J* = 12.9, 4.8 Hz, 1 H), 2.01 (s, *J* = 5.5 Hz, 3 H), 1.84 (t, *J* = 12.2 Hz, 1 H). ^13^C NMR (214 MHz, CD_3_OD) δ 177.33, 174.59, 169.62, 138.68, 134.21, 130.07, 130.03, 129.69, 129.66, 97.72, 71.86, 71.73, 70.63, 68.63, 54.11, 45.06, 41.69, 22.91, 22.85. HRMS (C_18_H_23_ClN_2_O_9_): calculated (MS-H): 445.10138, found: 445.10141.

### Sia-5

^1^H NMR (500 MHz, D_2_O) δ 7.73–7.46 (m, 3 H), 7.36 (dd, *J* = 13.8, 7.8 Hz, 1 H), 4.04 (d, *J* = 16.6, 7.5 Hz, 2 H), 3.93 (m, *J* = 11.8, 8.7 Hz, 2 H), 3.77 (d, *J* = 14.1, 2.7 Hz, 1 H), 3.52 (m, *J* = 13.9, 8.0 Hz, 2 H), 2.25–2.20 (dd, 1 H), 2.01 (s, *J* = 6.7 Hz, 3 H), 1.84 (t, *J* = 12.3, 5.9 Hz, 1 H). ^13^C NMR (214 MHz, CD_3_OD) δ 177.36, 175.61, 174.62, 174.48, 171.41, 169.26, 164.64, 163.49, 138.06, 138.02, 131.54, 131.50, 129.80, 126.94, 124.20, 119.42, 119.32, 115.41, 115.30, 97.71, 71.90, 71.54, 70.63, 68.54, 54.10, 44.98, 41.79, 26.25, 22.82. HRMS (C_18_H_23_FN_2_O_9_): calculated (MS-H): 429.13093, found: 429.13080.

### Sia-6

^1^H NMR (500 MHz, D_2_O) δ 8.97 (d, *J* = 2.2 Hz, 1 H), 8.51 (dd, *J* = 8.5, 2.2 Hz, 1 H), 7.74 (d, *J* = 8.5 Hz, 1 H), 4.15 (s, *J* = 6.4 Hz, 2 H), 4.04–3.94 (m, 2 H), 3.89 (d, *J* = 10.1 Hz, 1 H), 3.81–3.75 (t, 1 H), 3.58 (dd, *J* = 14.2, 2.8 Hz, 1 H), 3.45 (d, *J* = 9.1 Hz, 1 H), 3.31 (dd, *J* = 14.2, 7.2 Hz, 1 H), 2.18 (dd, *J* = 12.9, 4.8 Hz, 1 H), 2.02 (s, 3 H), 1.79 (t, *J* = 12.2 Hz, 1 H). ^13^C NMR (214 MHz, CD_3_OD) δ 177.39, 174.62, 171.39, 150.66, 148.51, 138.78, 136.19, 128.27, 121.07, 97.75, 71.87, 71.59, 70.58, 68.65, 54.11, 44.58, 41.74, 22.95. HRMS (C_19_H_24_N_4_O_13_): calculated (MS): 516.13399, found: 516.13208.

### Sia-8

^1^H NMR (500 MHz, D_2_O) δ 8.23 (s, 1 H), 7.27 (d, *J* = 9.0 Hz, 1 H), 6.63 (d, *J* = 9.0 Hz, 1 H), 6.29 (s, 1 H), 4.08–4.00 (m, 2 H), 3.96 (d, *J* = 10.1 Hz, 1 H), 3.90 (dd, *J* = 12.9, 5.7 Hz, 1 H), 3.80 (dd, *J* = 13.9, 2.9 Hz, 1 H), 3.64 (dd, *J* = 17.3, 6.3 Hz, 1 H), 3.49 (d, *J* = 9.7 Hz, 1 H), 3.39 (dd, *J* = 6.9 Hz, 4 H), 2.23 (dd, *J* = 12.9, 4.8 Hz, 1 H), 2.04 (s, 3 H), 1.86 (t, *J* = 12.2 Hz, 1 H), 1.17 (t, *J* = 7.0 Hz, 6 H). ^13^C NMR (214 MHz, CD_3_OD) δ 177.21, 174.52, 165.60, 163.94, 159.07, 154.51, 149.27, 132.60, 111.60, 110.16, 109.43, 97.70, 97.22, 71.90, 71.87, 70.26, 68.68, 54.12, 45.98, 44.50, 41.77, 22.84, 12.71. HRMS (C_25_H_33_N_3_O_11_): calculated (MS-H): 550.20368, found: 550.20361.

### Sia-9

^1^H NMR (500 MHz, D_2_O) δ 4.05–3.95 (m, 2 H), 3.95–3.87 (dd, 1 H), 3.74 (m, *J* = 10.7, 4.5 Hz, 1 H), 3.47 (dd, *J* = 14.4, 2.6 Hz, 1 H), 3.44–3.39 (d, 1 H), 3.22 (dd, *J* = 14.5, 6.6 Hz, 1 H), 2.20 (dd, *J* = 12.8, 4.6 Hz, 1 H), 2.04 (s, 3 H), 1.82 (dd, *J* = 12.1 Hz, 3 H), 1.69 (dd, *J* = 13.0 Hz, 2 H), 1.57 (dd, *J* = 12.4 Hz, 1 H), 1.31 (m, *J* = 24.3, 12.1 Hz, 2 H), 1.23–1.08 (m, 3 H). ^13^C NMR (214 MHz, CD_3_OD) δ 177.40, 174.40, 161.21, 129.80, 126.96, 97.74, 71.88, 71.37, 71.26, 68.76, 54.12, 44.67, 41.79, 34.69, 30.76, 30.73, 26.72, 26.05, 22.85, 21.30. HRMS (C_18_H_31_N_3_O_9_): calculated (MS): 433.20603, found: 433.20425.

### Sia-10

^1^H NMR (500 MHz, D_2_O) δ 7.40 (t, *J* = 7.4 Hz,2 H), 7.33 (d, *J* = 7.1 Hz, 3 H), 4.32 (s, 2 H), 4.05–3.95 (m, 2 H), 3.92 (d, *J* = 10.1 Hz, 1 H), 3.75 (m, *J* = 6.7 Hz, 1 H), 3.53 (dd, *J* = 14.2, 2.2 Hz, 1 H), 3.43 (d, *J* = 9.0 Hz, 1 H), 3.19 (dd, *J* = 14.3, 7.1 Hz, 1 H), 2.21 (dd, *J* = 12.9, 4.8 Hz, 1 H), 2.03 (s, 3 H), 1.82 (t, *J* = 12.2 Hz, 1 H). ^13^C NMR (214 MHz, CD_3_OD) δ 177.45, 174.42, 161.77, 141.28, 129.46, 128.20, 127.96, 97.74, 71.91, 71.44, 68.74, 54.10, 44.76, 41.80, 22.87. HRMS (C_19_H_27_N_3_O_9_): calculated (MS-H): 440.16690, found: 440.16693.

### Sia-11

^1^H NMR (500 MHz, D_2_O) δ 7.38 (s, 1 H), 7.29 (t, *J* = 8.0 Hz, 1 H), 7.17 (d, *J* = 8.0 Hz, 1 H), 7.11 (d, *J* = 7.9 Hz, 1 H), 4.06–3.96 (m, 2 H), 3.96–3.88 (d, 1 H), 3.84–3.76 (m, 1 H), 3.62–3.54 (dd, 1 H), 3.47 (d, *J* = 8.9 Hz, 1 H), 3.28 (dd, *J* = 14.2, 6.9 Hz, 1 H), 2.22 (dd, *J* = 12.9, 4.7 Hz, 1 H), 2.02 (s, 3 H), 1.83 (t, *J* = 12.2 Hz, 1 H). ^13^C NMR (214 MHz, CD_3_OD) δ 177.50, 174.48, 158.44, 142.70, 135.38, 130.95, 122.81, 119.49, 117.82, 101.39, 97.78, 71.99, 71.62, 71.06, 68.68, 54.14, 44.48, 41.79, 22.83. HRMS (C_18_H_24_ClN_3_O_9_): calculated (MS-H): 460.11228, found: 460.11246.

### Sia-12

^1^H NMR (500 MHz, D_2_O) δ 7.44 (s, 1 H), 7.37 (d, *J* = 8.7 Hz, 1 H), 7.11 (d, *J* = 8.6 Hz, 1 H), 4.07–3.97 (m, 2 H), 3.96–3.89 (d, 1 H), 3.81 (m, 1 H), 3.58 (dd, *J* = 12.5 Hz, 1 H), 3.47 (d, *J* = 8.9 Hz, 1 H), 3.28 (dd, *J* = 14.1, 6.8 Hz, 1 H), 2.21 (dd, *J* = 12.8, 4.3 Hz, 1 H), 2.02 (s, 3 H), 1.84 (t, *J* = 12.1 Hz, 1 H). ^13^C NMR (214 MHz, CD_3_OD) δ 177.53, 174.53, 158.24, 141.36, 133.16, 131.33, 125.48, 121.01, 119.28, 97.78, 72.00, 71.63, 70.98, 68.63, 54.15, 44.48, 41.79, 22.85. HRMS (C_18_H_23_Cl_2_N_3_O_9_): calculated (MS-H): 494.07331, found: 494.07340.

### Sia-13

^1^H NMR (500 MHz, D_2_O) δ 7.56 (d, *J* = 8.0 Hz, 1 H), 7.49 (d, *J* = 8.0 Hz, 1 H), 7.33 (t, *J* = 7.7 Hz, 1 H), 7.21 (d, *J* = 7.6 Hz, 1 H), 4.06–3.97 (m, 2 H), 3.93 (d, *J* = 10.2 Hz, 1 H), 3.82 (m, *J* = 6.1 Hz, 1 H), 3.59 (dd, *J* = 14.5, 2.5 Hz, 1 H), 3.48 (d, *J* = 9.0 Hz, 1 H), 3.31 (dd, *J* = 14.4, 6.6 Hz, 1 H), 2.21 (dd, *J* = 13.0, 4.6 Hz, 1 H), 2.04 (s, 3 H), 1.83 (t, *J* = 12.2 Hz, 1 H). ^13^C NMR (214 MHz, CD_3_OD) δ 177.47, 174.44, 155.87, 137.51, 130.23, 128.35, 124.53, 123.47, 97.80, 71.90, 71.39, 71.06, 68.70, 54.12, 44.55, 41.77, 22.84. HRMS (C_18_H_24_ClN_3_O_9_): calculated (MS-H): 460.11228, found: 460.11255.

### Sia-14

^1^H NMR (500 MHz, D_2_O) δ 7.38–7.30 (s, 1 H), 7.21 (d, *J* = 8.2 Hz, 1 H), 7.06 (d, *J* = 7.9 Hz, 1 H), 4.06–3.97 (m, 2 H), 3.91 (dd, *J* = 19.6, 9.3 Hz, 1 H), 3.80 (m, 1 H), 3.57 (dd, *J* = 12.4 Hz, 1 H), 3.46 (d, *J* = 8.8 Hz, 1 H), 3.28 (dd, *J* = 14.1, 6.7 Hz, 1 H), 2.28 (s, 3 H), 2.21 (dd, *J* = 12.8, 4.3 Hz, 1 H), 2.02 (s, 3 H), 1.83 (t, *J* = 12.2 Hz, 1 H). ^13^C NMR (214 MHz, CD_3_OD) δ 177.49, 174.48, 158.63, 140.23, 135.09, 132.01, 131.91, 130.26, 129.09, 120.61, 120.22, 118.82, 118.43, 97.77, 71.97, 71.56, 71.09, 68.67, 54.12, 41.79, 30.73, 22.84, 19.31, 19.25. HRMS (C_19_H_26_ClN_3_O_9_): calculated (MS-H): 474.12793, found: 474.12796.

### Sia-15

^1^H NMR (500 MHz, D_2_O) δ 7.35 (d, *J* = 8.4 Hz, 2 H), 7.27 (d, *J* = 8.4 Hz, 2 H), 4.06–3.97 (m, 2 H), 3.95–3.88 (d, 1 H), 3.80 (m, *J* = 6.4 Hz, 1 H), 3.61–3.55 (dd, 1 H), 3.46 (d, *J* = 8.9 Hz, 1 H), 3.29 (dd, *J* = 14.3, 6.8 Hz, 1 H), 2.21 (dd, *J* = 12.9, 4.7 Hz, 1 H), 2.02 (s, 3 H), 1.83 (t, *J* = 12.2 Hz, 1 H). ^13^C NMR (214 MHz, CD_3_OD) δ 177.44, 174.51, 158.61, 140.03, 129.73, 129.63, 127.90, 121.25, 115.35, 97.78, 71.98, 71.58, 71.03, 68.67, 54.17, 44.50, 41.78, 30.73, 22.84. HRMS (C_18_H_24_ClN_3_O_9_): calculated (MS-H): 460.11228, found: 460.11246.

### Sia-16

^1^H NMR (500 MHz, D_2_O) δ 7.24 (s, 2 H), 7.10 (s, 2 H), 4.06–3.97 (m, 2 H), 3.93 (d, *J* = 10.0 Hz, 1 H), 3.81 (m, 1 H), 3.59 (dd, *J* = 12.9 Hz, 1 H), 3.47 (d, *J* = 8.8 Hz, 1 H), 3.28 (dd, *J* = 14.1, 6.8 Hz, 1 H), 2.21 (dd, *J* = 12.6, 4.2 Hz, 1 H), 2.02 (s, 3 H), 1.83 (t, *J* = 12.1 Hz, 1 H). ^13^C NMR (214 MHz, CD_3_OD) δ 177.43, 174.59, 157.99, 143.79, 136.01, 122.19, 117.55, 97.80, 72.03, 71.82, 70.91, 68.66, 54.23, 44.54, 41.80, 22.84. HRMS (C_18_H_23_Cl_2_N_3_O_9_): calculated (MS-H): 494.07331, found: 494.07337.

### Sia-17

^1^H NMR (500 MHz, D_2_O) δ 7.65 (s, 1 H), 7.41 (d, *J* = 8.7 Hz, 1 H), 7.36 (d, *J* = 8.4 Hz, 1 H), 4.02 (m, *J* = 19.5, 10.3 Hz, 2 H), 3.96–3.89 (d, 1 H), 3.81 (m, 1 H), 3.60 (dd, *J* = 13.2 Hz, 21 H), 3.47 (d, *J* = 8.8 Hz, 1 H), 3.28 (dd, *J* = 13.6, 6.5 Hz, 1 H), 2.22 (dd, *J* = 8.6 Hz, 1 H), 2.02 (s, 3 H), 1.84 (t, *J* = 12.0 Hz, 1 H). ^13^C NMR (214 MHz, CD_3_OD) δ 177.50, 174.53, 158.19, 140.83, 132.84, 129.24, 129.10, 124.97, 124.68, 123.80, 123.69, 118.27, 97.78, 72.03, 71.79, 70.98, 68.67, 54.16, 44.55, 41.78, 22.84. HRMS (C_19_H_23_ClF_3_N_3_O_9_): calculated (MS-H): 528.09967, found: 528.09961.

### Sia-18

^1^H NMR (500 MHz, D_2_O) δ 7.81 (s, 2 H), 7.66 (s, 1 H), 4.02 (m, *J* = 18.8, 10.4 Hz, 2 H), 3.93 (d, *J* = 10.0 Hz, 1 H), 3.82 (m, 1 H), 3.62 (dd, *J* = 12.8 Hz, 1 H), 3.48 (d, *J* = 8.6 Hz, 1 H), 3.29 (dd, *J* = 13.8, 6.7 Hz, 1 H), 2.22 (dd, *J* = 8.9 Hz, 1 H), 2.02 (s, 3 H), 1.84 (t, *J* = 12.0 Hz, 1 H). ^13^C NMR (214 MHz, CD_3_OD) δ 177.46, 174.56, 157.94, 143.59, 133.14, 132.99, 125.50, 124.23, 118.94, 115.27, 97.78, 72.08, 70.92, 68.70, 54.22, 44.62, 41.82, 22.81. HRMS (C_20_H_23_F_6_N_3_O_9_): calculated (MS-H): 562.12602, found: 562.12586.

### Sia-19

^1^H NMR (500 MHz, D_2_O) δ 7.61 (d, *J* = 8.4 Hz, 2 H), 7.42 (d, *J* = 8.3 Hz, 2 H), 4.06–3.98 (m, 2 H), 3.94 (d, *J* = 10.1 Hz, 1 H), 3.81 (m, 1 H), 3.63–3.56 (dd, 1 H), 3.48 (d, *J* = 8.9 Hz, 1 H), 3.29 (dd, *J* = 14.1, 6.9 Hz, 1 H), 2.22 (dd, *J* = 12.9, 4.6 Hz, 1 H), 2.01 (s, 3 H), 1.84 (t, *J* = 12.1 Hz, 1 H). ^13^C NMR (214 MHz, CD_3_OD) δ 177.53, 174.59, 158.30, 144.90, 126.92, 126.90, 126.59, 125.32, 124.47, 119.14, 97.83, 71.99, 71.56, 70.95, 68.61, 54.19, 44.45, 41.79, 30.71, 22.86, 22.68. HRMS (C_19_H_24_F_3_N_3_O_9_): calculated (MS-H): 494.13864, found: 494.13866.

### Sia-20

^1^H NMR (500 MHz, D_2_O) δ 7.69 (d, *J* = 8.3 Hz, 1 H), 7.50–7.43 (m, 1 H), 7.37 (d, *J* = 8.0 Hz, 1 H), 7.08–7.02 (t, 1 H), 6.98 (t, *J* = 9.9, 2.7, 1.3 Hz, 1 H), 4.06–3.99 (m, 2 H), 3.94 (d, *J* = 10.1 Hz, 1 H), 3.83–3.78 (m, 1 H), 3.59 (dd, *J* = 14.4, 3.1 Hz, 1 H), 3.48 (d, *J* = 9.0 Hz, 1 H), 3.30 (dd, *J* = 14.4, 6.8 Hz, 1 H), 2.22 (dd, *J* = 12.9, 4.8 Hz, 1 H), 2.05 (s, 3 H), 1.84 (dd, *J* = 15.4, 9.0 Hz, 1 H). ^13^C NMR (214 MHz, CD_3_OD) δ 177.37, 174.71, 143.23, 141.80, 129.83, 126.90, 125.11, 124.76, 124.56, 111.89, 111.79, 111.76, 111.64, 104.61, 104.48, 104.36, 104.23, 97.80, 71.90, 71.21, 70.89, 68.48, 54.14, 44.44, 41.70, 22.87, 21.30. HRMS (C_18_H_23_F_2_N_3_O_9_): calculated (MS-H): 462.13241, found: 462.13262.

### Sia-21

^1^H NMR (500 MHz, D_2_O) δ 6.98–6.93 (m, 2 H), 6.67–6.60 (t, 1 H), 4.06–3.97 (m, 2 H), 3.93 (d, *J* = 7.9 Hz, 1 H), 3.84–3.78 (dd, 1 H), 3.62–3.57 (d, 1 H), 3.47 (d, *J* = 8.9 Hz, 1 H), 3.29 (dd, *J* = 14.3, 7.0 Hz, 1 H), 2.22 (dd, *J* = 12.8, 4.6 Hz, 1 H), 2.03 (s, 1 H), 1.86–1.81 (t, 1 H). ^13^C NMR (214 MHz, CD_3_OD) δ 177.64, 174.52, 165.30, 165.23, 164.16, 164.09, 158.11, 144.14, 102.09, 101.96, 97.79, 97.49, 97.36, 71.97, 71.33, 70.91, 68.48, 54.07, 44.27, 41.83, 22.84. HRMS (C_18_H_23_F_2_N_3_O_9_): calculated (MS): 463.14024, found: 463.13835.

### Sia-22

^1^H NMR (500 MHz, D_2_O) δ 7.91 (t, *J* = 12.4, 5.2 Hz, 2 H), 7.80 (d, *J* = 8.2 Hz, 1 H), 7.53 (m, *J* = 9.1, 5.3 Hz, 2 H), 7.47 (t, *J* = 7.8 Hz, 1 H), 7.40 (t, *J* = 7.1 Hz, 1 H), 4.03–3.94 (m, 2 H), 3.93–3.85 (d, 1 H), 3.77 (m, 1 H), 3.56 (d, *J* = 14.2 Hz, 1 H), 3.43 (d, *J* = 8.9 Hz, 1 H), 3.26 (dd, *J* = 14.3, 6.7 Hz, 1 H), 2.19 (dd, *J* = 12.9, 4.7 Hz, 1 H), 1.98 (s, 3 H), 1.80 (t, *J* = 12.2 Hz, 1 H). ^13^C NMR (214 MHz, CD_3_OD) δ 177.42, 174.49, 159.87, 135.75, 135.36, 129.38, 126.97, 126.93, 126.69, 125.61, 122.84, 121.55, 97.80, 74.37, 71.94, 71.49, 71.19, 68.73, 54.17, 44.76, 41.77, 22.88. HRMS (C_22_H_27_N_3_O_9_): calculated (MS-H): 476.16690, found: 476.16664.

### Sia-23

^1^H NMR (500 MHz, D_2_O) δ 4.10–3.97 (m, 2 H), 3.97–3.81 (m, 3 H), 3.73–3.58 (m, 1 H), 3.54–3.40 (dd, 1 H), 2.96 (s, 3 H), 2.23 (dd, *J* = 13.0, 4.8 Hz, 1 H), 2.07 (s, 3 H), 1.84 (t, *J* = 12.2 Hz, 1 H). ^13^C NMR (214 MHz, CD_3_OD) δ 177.66, 174.64, 97.83, 71.83, 71.14, 68.44, 54.03, 41.79, 22.95. HRMS (C_13_H_23_N_3_O_8_S): calculated (MS + K): 420.08429, found: 420.07712.

### Sia-24

^1^H NMR (500 MHz, D_2_O) δ 6.95 (d, *J* = 8.2 Hz, 1 H), 6.83 (s, *J* = 15.6 Hz, 1 H), 6.79 (dd, *J* = 8.0 Hz, 1 H), 6.05 (s, 2 H), 4.11–3.82 (m, 5 H), 3.69 (m, 1 H), 3.48 (d, *J* = 8.1 Hz, 1 H), 2.24 (dd, *J* = 12.9, 4.8 Hz, 1 H), 2.12–2.06 (s, 3 H), 1.86 (t, *J* = 12.2 Hz, 1 H). ^13^C NMR (214 MHz, CD_3_OD) δ 177.26, 174.48, 119.85, 109.34, 108.00, 102.95, 97.68, 71.84, 68.70, 54.05, 41.70, 22.88. HRMS (C_19_H_25_N_3_O_10_S): calculated (MS + Na): 510.11583, found: 510.11632.

### Sia-25

^1^H NMR (500 MHz, D_2_O) δ 7.98 (s, 3 H), 4.15–3.91 (m, 5 H), 3.71 (m, 1 H), 3.54 (dd, *J* = 8.8 Hz, 1 H), 2.25 (dd, *J* = 8.9 Hz, 1 H), 2.07 (s, 3 H), 1.87 (t, *J* = 11.8 Hz, 1 H). ^13^C NMR (214 MHz, CD_3_OD) δ 183.11, 177.05, 174.64, 143.45, 132.59, 132.43, 126.68, 125.41, 124.14, 123.44, 122.87, 117.48, 97.68, 72.10, 68.62, 54.21, 47.82, 41.67, 30.73, 22.83, 9.21. HRMS (C_20_H_23_F_6_N_3_O_8_S): calculated (MS + Na): 602.10077, found: 602.10176.

### Sia-26

^1^H NMR (500 MHz, D_2_O) δ 7.37–7.27 (m, 2 H), 7.23 (t, *J* = 8.1 Hz, 2 H), 4.10–3.81 (m, 5 H), 3.69 (m, 1 H), 3.49 (dd, *J* = 7.6 Hz, 1 H), 2.24 (dd, *J* = 13.0, 4.6 Hz, 1 H), 2.14–2.02 (s, 3 H), 1.87 (t, *J* = 25.8, 13.5 Hz, 1 H). ^13^C NMR (214 MHz, CD_3_OD) δ 182.85, 177.59, 174.70, 164.87, 162.22, 161.08, 127.94, 116.55, 116.20, 116.10, 97.83, 71.77, 71.48, 70.50, 68.46, 54.06, 41.63, 37.05, 31.72, 23.07, 23.02. HRMS (C_18_H_24_FN_3_O_8_S): calculated (MS + Na): 484.11658, found: 484.11682.

## Electronic supplementary material

Supplementary Information
