# Supplementary material for: On the use of abiotic sialic acids to attenuate cell inflammation
Source: Sci Rep. 2018 Nov 23;8:17320. doi: 10.1038/s41598-018-35477-2 (PMC6251910; doi:10.1038/s41598-018-35477-2)
Supplement: Supplementary file 1 — Supplementary Information [file 41598_2018_35477_MOESM1_ESM.pdf]

Electronic Supporting Information

On the use of abiotic sialic acids to attenuate cell inflammation

Zhongwei Xue,[a,#] Hu Zhao,[a,#] Rui Zhu,[a] Congcong Chen,[c] Hongzhi Cao,[c] Jiahuai Han,[b] and Shoufa Han[a,]*

[a]Department of Chemical Biology, College of Chemistry and Chemical Engineering, the Key Laboratory for Chemical Biology of Fujian Province, The MOE Key Laboratory of Spectrochemical Analysis & Instrumentation, and Innovation Center for Cell Biology, Xiamen University; [b]State key Laboratory of Cellular Stress Biology, Innovation Center for Cell Biology, School of Life Sciences, Xiamen University, Xiamen, 361005, China; [c]National Glycoengineering research center, Shandong University, Jinan, 250012, China

Tel: 86-0592-2181728; E-mail: shoufa@xmu.edu.cn

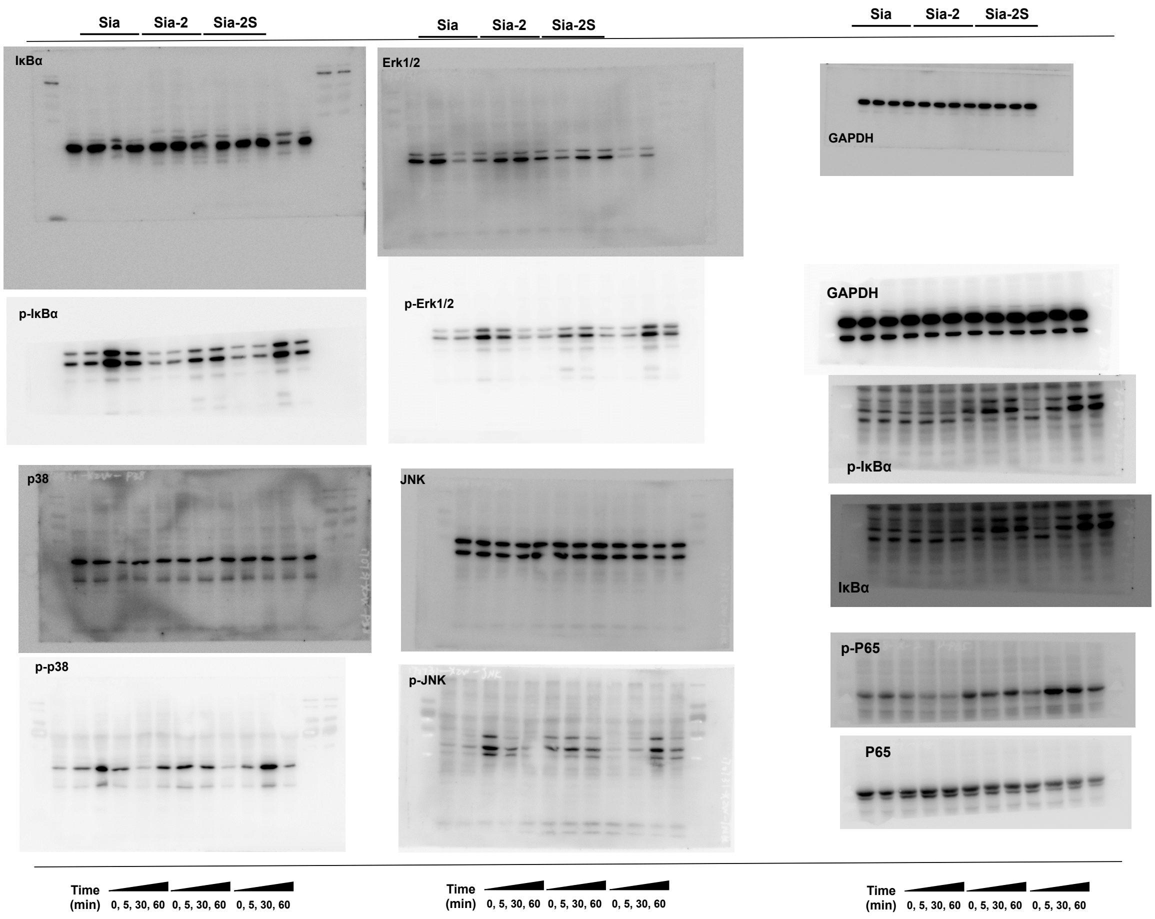

Figure S1. Effects of Sia-2 and Sia-2S on phosphorylation of key components of inflammation-relevant cell signaling pathways. Raw 264.7 cells were first cultured for 24 h in DMEM spiked with Sia (0.5 mM), Sia-2 (0.5 mM) or Sia-2S (0.5 mM) and stimulated with LPS (100 ng/mL) for0, 5, 30, and 60 min. The cell samples were respectively added to 1.2 x SDS-PAGE loading buffer, resolved on 10% SDS-PAGE gels, transferred to nitrocellulose, and blocked with 5% bovine serum albumin in PBST for 1 h at room temperature. The blocked membrane was incubated with specific first antibodies in blocking buffer overnight at 4 ℃ and washed with PBST (3 x 10 min per wash). Followed by HRP-conjugated second antibodies in blocking buffer for 1 h at room temperature. Then the membrane was washed with PBST (3 x 10 min per wash), and developed using Immobilion Western Chemilum HRP Substrate (Merck).
